# Uncovering novel mechanisms of chitinase-3-like protein 1 in driving inflammation-associated cancers

**DOI:** 10.1186/s12935-024-03425-y

**Published:** 2024-07-27

**Authors:** Yan Fan, Yuan Meng, Xingwei Hu, Jianhua Liu, Xiaosong Qin

**Affiliations:** 1 Department of Laboratory Medicine, Liaoning Clinical Research Center for Laboratory Medicine, Shengjing Hospital of China Medical University, Shenyang, Liaoning 110122 China; 2 Liaoning Clinical Research Center for Laboratory Medicine, Shenyang, Liaoning Province China

**Keywords:** Chitinase-3-like protein 1, Chronic inflammation, Tumor progression, Tumor-associated inflammation

## Abstract

Chitinase-3-like protein 1 (CHI3L1) is a secreted glycoprotein that is induced and regulated by multiple factors during inflammation in enteritis, pneumonia, asthma, arthritis, and other diseases. It is associated with the deterioration of the inflammatory environment in tissues with chronic inflammation caused by microbial infection or autoimmune diseases. The expression of CHI3L1 expression is upregulated in several malignant tumors, underscoring the crucial role of chronic inflammation in the initiation and progression of cancer. While the precise mechanism connecting inflammation and cancer is unclear, the involvement of CHI3L1 is involved in chronic inflammation, suggesting its role as a contributing factor to in the link between inflammation and cancer. CHI3L1 can aggravate DNA oxidative damage, induce the cancerous phenotype, promote the development of a tumor inflammatory environment and angiogenesis, inhibit immune cells, and promote cancer cell growth, invasion, and migration. Furthermore, it participates in the initiation of cancer progression and metastasis by binding with transmembrane receptors to mediate intracellular signal transduction. Based on the current research on CHI3L1, we explore introduce the receptors that interact with CHI3L1 along with the signaling pathways that may be triggered during chronic inflammation to enhance tumorigenesis and progression. In the last section of the article, we provide a brief overview of anti-inflammatory therapies that target CHI3L1.

## Introduction

Inflammation is a vital defense mechanism triggered in response to injuries or harmful stimuli, and plays a key role in facilitating the repair of damaged tissues and restoring the body’s internal environment of the body to a state of equilibrium [1]. When inflammatory factors persist, they can lead to various diseases, including conditions marked by excessive stress responses, tissue damage, angiogenesis, fibrosis, and steatosis, and may even induce cancer development at the site of inflammation site [2, 3]. Currently, tumor-associated inflammation is recognized as the seventh major aspect for tumor progression following enhanced proliferative potential, resistance to growth inhibition, evasion of programmed cell death, uncontrolled replication, activation of angiogenesis, and initiation of invasion and metastasis [4, 5].

Inflammation contributes to cancer progression via endogenous and exogenous pathways. The former represents the primary mechanism by which inflammation promotes tumor growth. Various gene mutations or epigenetic changes lead to the inactivation of tumor suppressor signaling and activation of carcinogenic pathways; consequently, inflammatory mediators are produced in the absence of overt inflammation, thereby creating a tumor-inflammatory microenvironment. In the second pathway, factors such as viruses or other microbial infections contribute to tumor progression [5, 6].

Recently, a growing body of research has indicated that chitinase-3-like protein 1 (CHI3L1; also known as breast regression protein 39 [BRP-39] in mice) may serve as an important marker of chronic inflammation and subsequently promote tumor progression [7–10]. First, it exacerbates oxidation-induced DNA damage, thereby elevating the risk of cells transforming into cancerous forms [11, 12]. Second, it participates in mediating autoimmune diseases and inflammation caused by microbial infections, among other factors [13, 14]. CHI3L1 can recruit immune cells, influence cell apoptosis, maintain cell proliferation signals, activate invasion, and initiate tumor development and metastasis under inflammatory conditions. In solid tumors, CHI3L1 is mainly produced by cancerous and tumor-associated immune cells that infiltrate the vicinity of the tumor tissue. Moreover, it serves as an important potential regulator within the inflammatory tumor microenvironment [15–17].

**Fig. 1:**
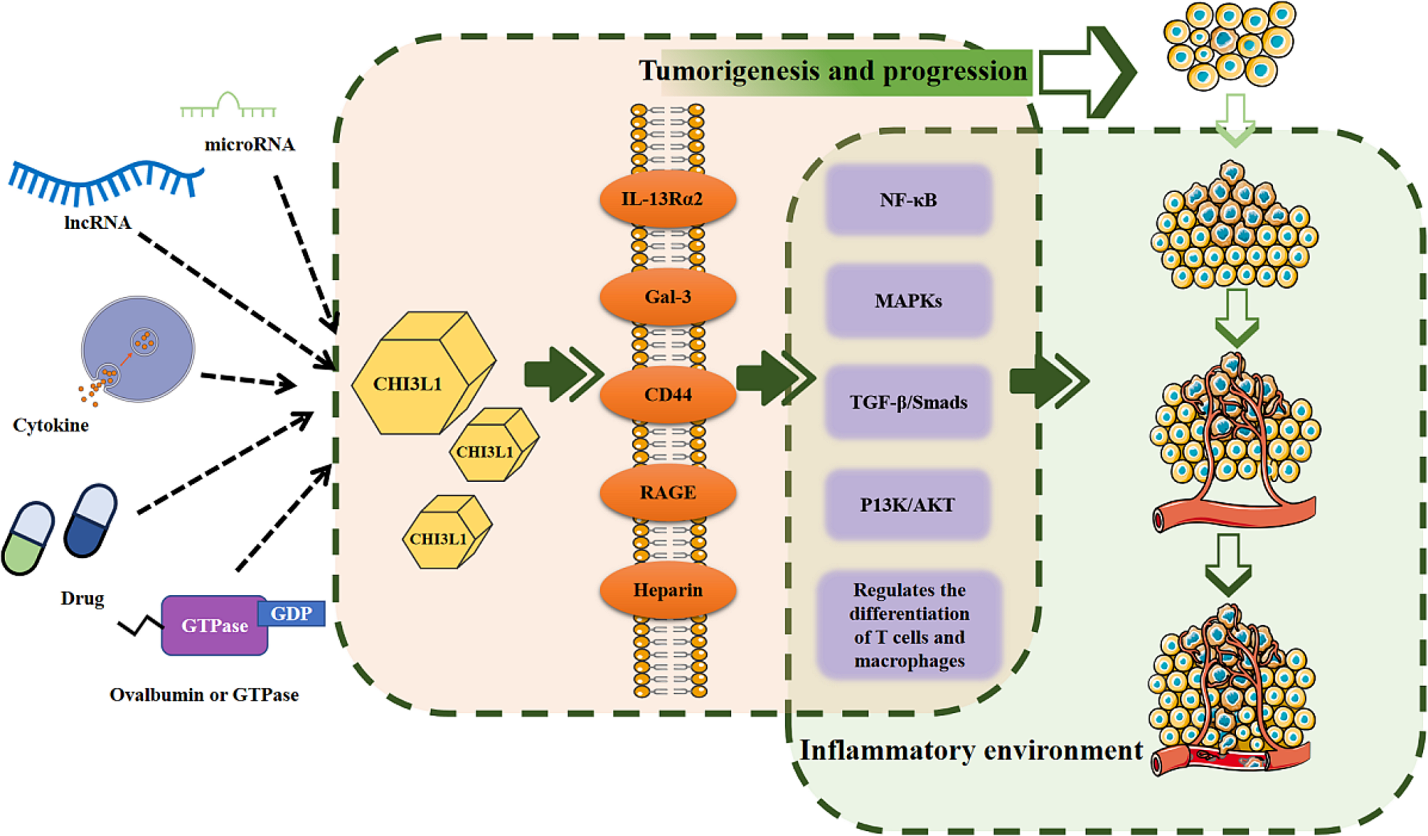
Factors regulating CHI3L1, and the role of CHI3L1 receptors and signaling pathways in cancer progression. Note: CD44, cluster of differentiation 44; CHI3L1, chitinase-3-like protein 1; Gal-3, galectin-3; IL-13Rα2, interleukin-13 receptor subunit alpha-2; lncRNA, long noncoding RNA; MAPK, mitogen-activated protein kinase; NF-κB, nuclear factor kappa-light-chain enhancer of activated B cells; RAGE, receptor for advanced glycation end products; TGF, transforming growth factor

The upregulation of CHI3L1 in the body is associated with various types of cancer [18], and the interplay between CHI3L1 and other inflammatory factors is an active research area. The inflammatory response is a complex biological process that entails mutual regulation among multiple inflammatory factors. Therefore, delving into the interaction mechanism between CHI3L1 and other inflammatory factors can benefit our understanding of the mechanisms underlying inflammation response and tumor development. This review focuses on recently significant reported molecular mechanisms of CHI3L1 and inflammation-associated tumours, which provides new ideas to explore the development of inflammation-associated tumours (Fig. 1).

## Structure and properties of CHI3L1

CHI3L1 comprises 10 exons and is located within an 8-kb DNA fragment on chromosome 1q32.1. The CHI3L1 polypeptide chain consists of 383 amino acids, with a relative molecular weight of approximately 40 kDa. The three N-terminal amino acids of the peptide chain are tyrosine (Y), lysine (K), and leucine (L); hence, CHI3L1 is also known as YKL-40 [19]. Humans co-express four chitinase-like proteins (CLPs): CHI3L1, CHI3L2 (also known as YKL-39), oviductin (OVGP1), and stabilin-1 interacting chitinase-like protein (SI-CLP) [20]. OVGP1 plays a critical role in fertilization and early embryonic development [21]. The levels of SI-CLP levels are elevated in peripheral blood monocytes from patients with joint inflammation [22]. In recent years, it has been found that SI-CLP was discovered to interferes with the protumor effect of tumor-associated macrophages, and it has been identified as a potential targeting agent for tumor treatment [23]. YKL-39 is present only in humans and plays a role in chronic inflammatory and neurodegenerative diseases; its proangiogenic role in cancer was discovered after that of CHI3L1 [24, 25]. Among chitinases, CHI3L1 is the most extensively studied in relation to cancer; however, its role in disease has not been fully elucidated, primarily owing to its unique crystalline structure [26].

Crystal diffraction studies have revealed that CHI3L1 consists of two structural domains: an eight-stranded (β/α) triosephosphate isomerase (TIM) barrel domain and an α + β spherical structural domain consisting of six antiparallel β-strands and an α-helix, which is inserted into the β7-strand of the (β/α)_8_ TIM barrel structure, forming a bispherical structural domain [27]. Mammalian CHI3L1 is highly homologous and structurally similar to the 18-glycoside hydrolase family that has a (β/α) 8-TIM barrel domain containing glutamate and aspartate residues, which is critical for substrate hydrolysis in the catalytic center. Variations in the glutamic acid residue in the TIM barrel domain of CHI3L1 cause in impaired chitinase activity [28]. Additionally, a 43-residue carbohydrate-binding cleft has been identified in this TIM barrel structural domain, which can participate in ligand recognition [27]. Therefore, CHI3L1 lacks catalytic activity despite its chitin-binding ability. This structural complexity suggests that CHI3L1 can act as a binding cytokine with growth factor properties and play a crucial role in regulating innate defenses and inflammatory responses [29–31].

Studies have shown that several factors can regulate the expression of CHI3L1 expression in the body, including non-coding RNA, various cytokines, growth factors, and the tumor necrosis factor (TNF) (Table 1).

**Table 1:** Regulatory factors that affect CHI3L1 expression

Factors		Targeted cell		References
**MicroRNAs**	miRNA-24	AoSMC, RAW264.7 cells,	↓	[32, 33]
	miRNA-125a-3p	A549, H460 cell lines	↓	[34]
	miRNA-101	human glioblastoma stem cells	↓	[35]
	miRNA-149-5p	BEAS-2B cells	↓	[36]
	miRNA26a/b-5p	HEC-1 A, HEC-1B, RL95-2 cell lines	↓	[37]
	miRNA-302c-3p	human endometrial carcinoma cell	↓	[38]
**LncRNAs**	LncRNA NEAT1	HEC-1 A, HEC-1B, RL95-2 cell lines	↑	[37]
**Cytokines**	IL-1 and cytokines of the IL-6 family	U373-MG cells	↑	[39]
	IL-4, IL-17, IL-18	human keratinocytes	↑	[40]
	IFN-γ	fibroblasts	↑	[41]
	TNF-α	alveolar macrophages	↑	[42]
**Drugs**	Vitamin D		↓	[43]
	csDMARD + infliximab		↓	[44]
	Dexamethasone	BEAS-2B cells	↓	[45]
	Bortezomib		↓	[46]
**OVA/GTPase**	Ovalbumin	mouse tracheal epithelial cells	↑	[47]
	Rab37	RAW264.7, T cell lines	↑	[48]

Note: csDMARD, conventional synthetic disease-modifying antirheumatic drug; IFN, interferon; IL, interleukin; lncRNA, long noncoding RNA; TNF, tumor necrosis factor

## Receptors and binding partners of CHI3L1 involved in cancer progression

**Fig. 2:**
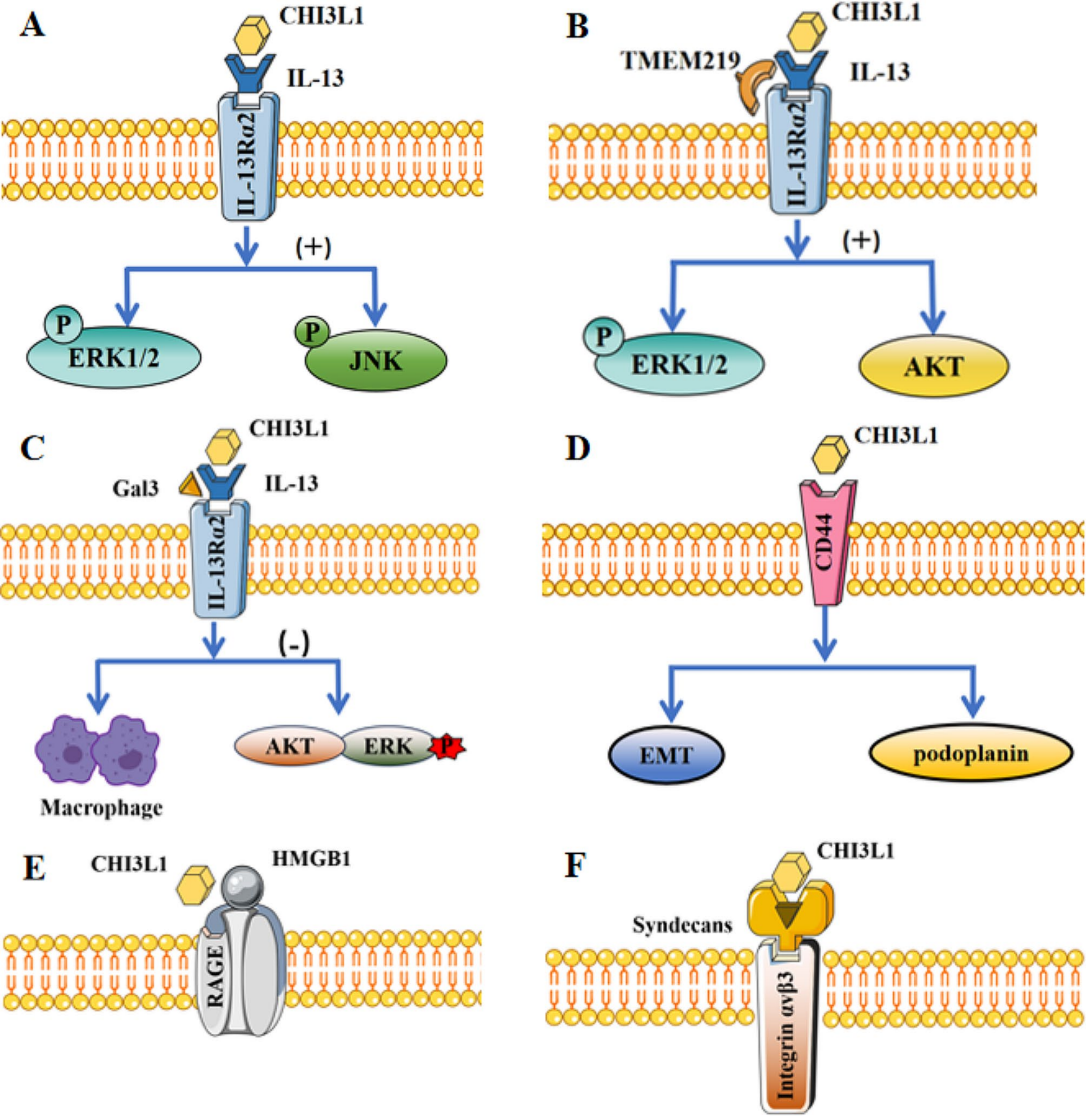
Receptors associated with CHI3L1 in cancer progression. Note: (**A**) CHI3L1 forms a multimer with IL-13–IL-13Rα2, activating the MAPK/ERK1/ERK2/JNK signaling pathway. (**B**) TMEM219 enhances MAPK/ERK1/ERK2 and AKT signaling when CHI3L1 interacts with IL-13–IL-13Rα2. (**C**) Gal-3 interacts with CHI3L1–IL-13–IL-13Rα2, impairing the AKT and ERK signaling pathways and inducing macrophage accumulation in fibrotic tissues. (**D**) CHI3L1 interacts with CD44, mediating the epithelial–mesenchymal transition of gastric cancer cells, thereby promoting the progression of gastric cancer. (**E**) CHI3L1 binds to RAGE, inhibiting the cytotoxic effect of immune cells on tumor cells in the tumor microenvironment, thereby promoting breast cancer metastasis. (**F**) CHI3L1 stimulates angiogenesis by interacting with acetylheparin sulfate chains and integrins, ensuring sufficient nutrients during tumor cell growth

CD44, cluster of differentiation 44; CHI3L1, chitinase-3-like protein 1; EMT, epithelial–mesenchymal transition; ERK, extracellular signal-regulated kinase; Gal-3, galectin-3; HMGB1, high mobility group box 1; IL-13, interleukin-13; IL-13Rα2, interleukin-13 receptor subunit alpha-2; JNK, c-Jun N-terminal kinase; MAPK, mitogen-activated protein kinase; TMEM219, transmembrane protein 219; RAGE, receptor for advanced glycation end products.

Recent studies suggest that the involvement of CHI3L1 in inflammation-associated cancer progression may be related to the cell surface target receptors. These target receptors include the interleukin-13 receptor α2 (IL-13Rα2), galectin-3 (Gal-3), chemoattractant receptor-homologous molecule expressed on T helper type 2 cells (CRTH2), cluster of differentiation 44 (CD44), and the receptor for advanced glycation end products (RAGE). Here, we elucidate the mode of action of CHI3L1, either through its own domain or via its binding to the relevant receptors and receptor partners. Additionally, we summarize the potential binding sites of CHI3L1 involved in regulating the cell cycle to promote cancer and its role as a messenger to activate multiple downstream signaling pathways (Fig. 2).

### CHI3L1/IL-13Rα2

IL-13Rα2 is a transmembrane protein with three distinct phenotypes: intracellular, membrane-bound, and soluble. It serves as a key target in numerous cancer treatment approaches. The IL-13Rα2 polypeptide effectively blocks the interaction/signal transduction between the original ligand and receptor [49], making it a widely recognized target receptor for CHI3L1. CHI3L1 exhibits high-affinity binding to the extracellular domain of IL-13Rα2 in lung, gastric, and breast cancer cells [50], subsequently triggering extracellular signal-regulated kinase (ERK)1/2 and c-Jun N-terminal kinase (JNK) phosphorylation to activate the mitogen-activated protein kinase (MAPK) signaling pathway, thereby promoting cancer progression [15, 51–55].

CHI3L1 can also form multimeric complexes with other binding partners of IL-13Rα2. IL-13 serves as a decoy ligand for IL-13Rα2 and does not compete with CHI3L1 for the IL-13Rα2 binding site with CHI3L1. Instead, CHI3L1 forms complexes with IL-13 and IL-13Rα2 to modulate immune responses, apoptosis, oxidative damage, fibrosis, and tissue repair (Fig. 2A) [56]. Lee et al. [57] used yeast two-hybrid technology to identify transmembrane protein 219 (TMEM219) as the binding protein of IL-13Rα2. TMEM219 demonstrates a strong interaction with the IL-13Rα2–CHI3L1 complex. In the absence of TMEM219 or IL-13Rα2, the ability of CHI3L1 to induce MAPK/Erk and Akt/PKB signaling is diminished, leading to increased apoptosis and oxidative damage (Fig. 2B).

### CHI3L1/Gal-3

Gal-3 is a multifunctional, water-soluble, carbohydrate-binding protein [58] that plays a pivotal role in numerous pathophysiological processes [59]. Gal-3 is expressed in the cytoplasm, nucleus, and cell surface. During the progression of chronic liver disease, Gal-3 exacerbates extracellular matrix synthesis and fibrosis formation through autocrine signaling [60]. Gal-3 can also form a multimeric complex with CHI3L1 and IL-13Rα2, blocking the binding of TMEM219 to IL-13Rα2 (Fig. 2B). This competitive binding modality activates the Wnt/β-linker pathway in macrophages while weakening the antiapoptotic effects of Erk and Akt signaling. Furthermore, the interaction exacerbates responses in the hereditary Hermansky–Pudlak syndrome, which is characterized by severe tissue damage and lung fibrosis (Fig. 2C) [61].

Orthogonal structure screening for membrane-associated binding proteins of CHI3L1 in glioblastoma predicted that Gal3BP, encoded by LGALS3BP, competes with Gal-3 for the same binding site on CHI3L1. Gal-3BP counteracts the tumor-promoting effect of CHI3L1 by binding to Gal-3 with a higher affinity [62].

### CHI3L1/CRTH2

CRTH2 is a G protein-coupled receptor present on T helper type 2 cells that mediates lymphocyte and eosinophil chemotaxis and contributes to the development of inflammation [63]. An increase in CHI3L1 expression has been observed in conjunction with an increase in CRTH2 levels in mice with renal injury, with BRP-39 potentially activating fibrosis progression after renal injury via the CRTH2 receptor [64]. In the context of pulmonary fibrosis, CHI3L1 interacts with CRTH2 on human monocytes, inducing macrophage polarization and regulating disease progression [65]. Recent studies have shown that CHI3L1 inhibits the β-catenin signaling pathway by binding to the CRTH2 receptor on neural stem cells in neuromyelitis optica spectrum disorders, subsequently leading to abnormalities in neurogenesis and cognitive dysfunction [66].

### CHI3L1/CD44

CD44 is a transmembrane protein present on the membrane surfaces of most vertebrate cells that plays a role in regulating cell growth, survival, proliferation, and migration [67]. Functionally, CD44 and CLPs are involved in neural development, homeostasis, repair, and injury responses in the nervous system. CD44 exists in four isoforms after splicing: CD44s, CD44v3, CD44v6, and CD44v9 [68]. The CD44v3 subtype binds to CHI3L1 in a concentration-dependent manner, promoting the epithelial–mesenchymal transition of gastric cancer cells (Fig. 2D) [69]. Shan et al. [70] found that following excessive acetaminophen use in patients and mice, CHI3L1 binding to the CD44 receptor on macrophage cell surfaces induced podophyllin expression, which can inhibit DNA synthesis and further exacerbate liver injury. In the context of glioma progression, CHI3L1 is released into the tumor microenvironment and interacts with CD44 expressed on tumor-associated macrophages, thereby activating the downstream PI3K/Akt signaling pathway and contributing to M2 macrophage polarization [71].

### CHI3L1/RAGE

RAGE is a single transmembrane receptor with multiple ligands that mediates the binding of several molecules, including advanced glycation end products (AGEs) [72]. The intact structure consists of three main regions: the extracellular, cytosolic, and intracellular fragments. The extracellular fragment interacts with different ligands, whereas the intracellular fragment can activate downstream signaling pathways [73]. High mobility group box 1 (HMGB1), a classic ligand of RAGE, has been extensively reported to promote cancer cell progression, particularly in gastrointestinal cancer [74–76]. Darwich et al. [77] found that CHI3L1 also binds to the RAGE receptor on the surface of natural killer (NK) cells, inhibiting their cytotoxicity (antibody-dependent cell-mediated cytotoxicity). This inhibition enhances the ability of tumor cells to evade the immune system, leading to a sudden decline in the clinical efficacy of trastuzumab in patients with metastatic human epidermal growth factor receptor 2 (HER2) breast cancer (Fig. 2E).

### CHI3L1/syndecans

The C-terminal region of CHI3L1 contains a multi-base amino acid cluster, with key recognition sites at K337, K342, and arginine (R)344. Interestingly, none of these three mutants efficiently bind to heparin [78]. CHI3L1 can induce the coordination of other heparin-like molecules, such as heparan sulfate chains (syndecans), with neighboring membrane proteins to trigger intracellular signaling pathways (Fig. 2F) [79]. CHI3L1 induces angiogenesis by interacting with the syndecan-1 receptor and integrin αvβ3 on the surface of vascular endothelial cells. Additionally, syndecan-4, a member of the syndecan family, mediates the signaling pathway that triggers MAPK, ultimately promoting angiogenesis [80, 81]. In studies on glioblastoma, CHI3L1 has been found to stimulate angiogenesis by inducing a coordinated interaction between syndecan-1 and integrin αvβ5 on the surface of glioma cells [82].

## CHI3L1 promotes signaling pathways associated with oncogenic inflammatory responses

Although not all inflammation increases the risk of cancer (for example, some types of skin inflammation have a tumor suppressor effect [83]), up to 20% of all cancers worldwide are associated with inflammation caused by chronic infections or autoimmune diseases [84]. Examples include inflammatory bowel disease and colorectal cancer, human papillomavirus and cervical cancer, gastritis and gastric cancer caused by *Helicobacter pylori* infection, and schistosomiasis-induced cystitis and bladder cancer [85–89]. Inflammation-mediated events, such as the production of intracellular reactive oxygen species (ROS), the release of growth factors, and alterations in signal transduction pathways, precede or accompany tumor development [90, 91]. CHI3L1 plays similar roles in processes associated with the development of chronic inflammation and cancer (Table 2). At normal physiologic concentrations in the body, CHI3L1 inhibits inflammatory cell apoptosis to prevent lung and liver tissue damage from excessive inflammatory responses [92, 93]. In contrast, CHI3L1 expression is elevated in the serum of patients with progressive inflammatory diseases or malignant cancers, where the inhibition of inflammatory cell apoptosis promotes disease progression.

Whether promoting inflammatory progression to induce tumorigenesis or maintaining the inflammatory microenvironment for tumor growth, CHI3L1 plays a crucial role in multiple stages of progressive tumorigenesis. Upon binding to its receptor, CHI3L1 activates several intracellular signaling pathways, including the nuclear factor kappa-light-chain enhancer of activated B cells (NF-κB), MAPKs, transforming growth factor (TGF)-β/Smads, and P13K/AKT. The reprogramming of immune cells in the inflammatory microenvironment promotes the occurrence and development of inflammation-associated cancers.

**Table 2:** The “parallels” of CHI3L1 in inflammation and tumor progression

Role of CHI3L1	Effect on the progression of inflammation	Effect on tumor progression
**Cell proliferation**	Airway remodeling and aggravation of bronchitis [94]	Cancer cell growth [17]
**Angiogenesis**	Vascular lesions in inflamed tissues [95]	Accelerated tumor growth and distant metastasis [96, 97]
**Immune cell activation and recruitment**	Aggravation of inflammation and exacerbation of tissue damage [98, 99]	Inhibition of antitumor activity/deterioration of the tumor microenvironment [71, 100–103]
**Proinflammatory factor secretion**	Mediates inflammation caused by microbes [104]	Increases the invasive ability of cancer cells [103, 105]
**Extracellular matrix remodeling**	Joint destruction in arthritis [106]	Tumor invasion and metastasis [107, 108]

### CHI3L1 and colorectal cancer

Colorectal cancer is the third most common cancer worldwide, and the risk of developing the disease increases in patients with inflammatory bowel disease [109, 110]. In long-standing ulcerative colitis (UC), the severity of colonic inflammation is an important determinant of the progression to colorectal cancer [111, 112]. The damage and apoptosis of colon epithelial cells lead to the destruction of the mucosal barrier, which is an early event in the development of chronic intestinal inflammation and promotes the continuation of carcinogenesis. ROS contribute to the initiation of dysplasia in inflammatory bowel disease. Excessive ROS accumulation can induce protein dysfunction and DNA damage, which may lead to gene mutations and cell apoptosis associated with colitis–colorectal cancer transformation [113]. CHI3L1 does not increase ROS production in colon cancer cells (HT-29) under normal conditions; however, it increases ROS content in HT-29 cells under oxidative stress conditions. Consequently, the intestinal cell line becomes more susceptible to oxidative stress [114]. Imbalances in the gut microbiota can also damage the intestinal mucosa, leading to chronic tissue inflammation, the release of carcinogenic mediators, and an increased risk of colitis-associated colon cancer. Popov et al. proposed three theories regarding microbial involvement in the disease: the α-bug hypothesis, the driver–passenger hypothesis, and the common ground hypothesis [111]. The α-BUG hypothesis suggests that damage in the colon epithelium cells is directly caused by bacteria, especially enterotoxigenic *Bacteroides fragilis* (ETBF) at concentrations > 1 × 10^9^ CFU per gram of feces. Intestinal inflammation and even colon tumors can be observed when ETBF colonizes the intestinal tract of mice for more than four weeks [115]. The driver–passenger hypothesis posits that after the initial bacteria attack the lumen, other opportunistic pathogens start to grow and attack the gut, causing an ecological imbalance. In the common basis hypothesis, exogenous and endogenous factors trigger a “leaky gut” that results in transcellular hyperpermeability and bacterial infiltration. The process may lead to inflammation and subsequent cancer development in susceptible individuals [116].

As previously mentioned, owing to its structural variation, CHI3L1 can only bind but not hydrolyze chitin. The binding of CHI3L1 to bacteria through chitin-binding protein-21 (CBP21) or the chitin complex promotes bacterial adhesion and internalization without inducing bacterial hydrolysis [117, 118]. Moreover, the gene encoding CBP21, a chitin-binding protein, has been detected in most chitinase-producing microorganisms, suggesting the potential binding ability of CHI3L1 to bacteria [117]. Adherent-invasive *Escherichia coli* (AIEC) invades the intestinal tract by binding chitin-binding domain type 3 (CBD3), which contains amino acid variations in the N-glycosylated CHI3L1 overexpressed in colonic cells [118]. In mice infected with AIEC, as CHI3L1 expression increased significantly, the strain gradually invaded the colon, leading to the exacerbation of colonic inflammation [118].

CHI3L1 also enhances bacterial adhesion and invasion in the gut, depending on the strain. CHI3L1 can increase the number of pathogenic bacteria (*Salmonella typhimurium* and enteropathogenic *E. coli* [EPEC]) and opportunistic pathogens (AIEC) that attach to or invade the host in a dose-dependent manner. However, CHI3L1 did not promote the host invasion of the nonpathogenic *E. coli* strains DH10B and DH5α [119]. Proinflammatory cytokines, such as IL-1β and TNF-α, are key mediators for the development and progression of colitis-associated colon cancer [120]. IL-6 deficiency can reduce the formation and growth of colon cancer cells, and the effect is mediated by the transcription factor STAT3 [121]. CHI3L1 could also weaken the intestinal resistance to *S. typhimurium* and AIEC in mice at the early stage of infection, cooperate with IL-6 to activate the inflammatory transcription factor STAT3, and amplify colonic inflammation caused by bacterial invasion [122].

Canonical Wnt/β-catenin, p38 MAPK, NF-κB, and JAK/STAT pathways are extensively activated in colitis-related cancers, with NF-κB and STAT in particular being hyperactive throughout colitis and colitis-associated colon cancer [123]. In resting cells, NF-κB forms a complex with its specific inhibitory unit, IκB, which is retained in the cytosol in its inactive form. When cells are stimulated (via microbial and viral infections or proinflammatory cytokine responses), the activation of the IκB kinase (IKK) complex phosphorylates IκB, which exposes the nuclear localization site of NF-κB and enters the nucleus; this mechanism enables the activation of the transcription of target gene sets and mediation of various biological functions [124]. Ikkβ-dependent NF-κB activation promotes myeloid cells to produce the proinflammatory mediators IL-1β, ICAM, IL-6, MIP-2, and KC, leading to the proliferation of intestinal cells and tumor occurrence. This serves as a key pathway underlying the relationship between inflammation and tumors [125]. The expression of *CHI3L1* mRNA is elevated in the intestinal mucosa of patients with dysplastic and active UC, and patients with UC with distal neoplastic lesions exhibit increased CHI3L1 expression (even 20-fold) compared to healthy subjects. In SW480 cells stimulated by recombinant CHI3L1 in vitro, CHI3L1 significantly activated a limited set of genes. Among them, the intracellular (in colon epithelial cells) NF-κB pathway was specifically activated through CHI3L1–myd88/IRAK1 molecules. Secretion of the downstream inflammatory factors IL-8 and TNF-α is also dependent on NF-κB activation [126]. Sporadic colorectal cancer is a prevalent form, accounting for > 90% of colorectal cancer cases [109]. In contrast to the unique “mucosa–dysplasia–carcinoma” sequence implicated in the pathogenesis of colitis-associated colon cancer, sporadic colorectal cancer demonstrates a well-defined “mucosa–adenoma–carcinoma” process. In patients with sporadic colorectal cancer, the tumor suppressor adenomatous polyposis coli (*APC*) gene is mutated early to activate the canonical Wnt/β-catenin signaling pathway, whereas in patients with colitis-associated colon cancer, *APC* mutations occur late in cancer progression [127]. Low-dose purified CHI3L1 can continuously activate the Wnt/β-catenin pathway and trans-nucleic acid translocation in colon cancer epithelial cells, which has a direct, rather than secondary, effect on inflammation-associated colorectal cancer [128].

**Fig. 3:**
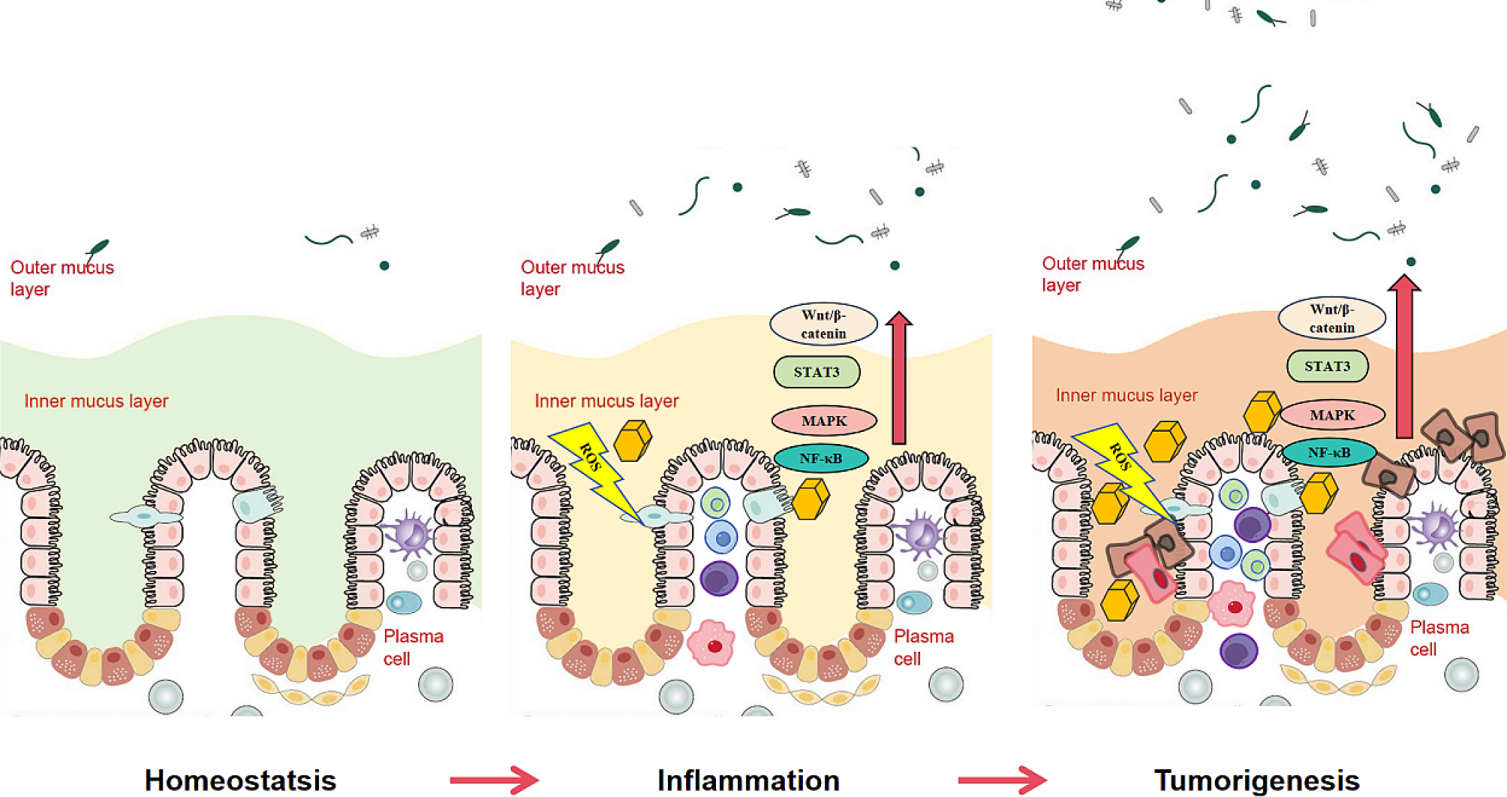
Cellular and molecular mechanisms underlying the development of CHI3L1 and colitis-associated cancer

Macrophages under hypoxic conditions in the tumor growth center are recognized as a decisive factor in colon cancer progression. Macrophage recruitment in the tumor microenvironment can help tumor cells achieve immune escape and promote growth and distant metastasis [129]. CHI3L1 significantly increases the proliferation of colon cancer cells and the secretion of IL-8 and monocyte chemoattractant protein-1 (MCP-1) by upregulating the phosphorylation of ERK1/2 and JNK and promoting the chemotaxis of macrophages to the tumor microenvironment and angiogenesis [103]. Cetuximab can specifically bind to epidermal growth factor (EGF) receptors on the surface of various tumor cells and inhibit cancer cell growth. It is the initial treatment option for metastatic colon cancer [130, 131]. However, unfortunately, not all patients with colon cancer are sensitive to cetuximab [132, 133]. CHI3L1 in the plasma of patients with metastatic colon cancer is a poor prognostic marker. CHI3L1 can significantly reduce the sensitivity of cells to cetuximab and promote the proliferation of colon cancer cells by downregulating the expression of the tumor suppressor gene *p53* and upregulating epidermal growth factor receptor (EGFR) expression; this suggests that CHI3L1 plays a key role in guiding individualized treatment with cetuximab [134]. Therefore, CHI3L1 may be closely linked to the chronic inflammation that mediates neoplastic transformations in the colon epithelium. The above evidence establishes that CHI3L1 is involved in the development and progression of colorectal cancer (Fig. 3).

### CHI3L1 and lung cancer

Lung cancer is the most common cancer [109]. In addition, lung inflammatory diseases such as asthma, chronic obstructive pulmonary disease (COPD), and pulmonary fibrosis have a high mortality rate and can promote the occurrence and development of lung cancer [135–137]. CHI3L1 is a potential biomarker for the prediction of local and systemic inflammation-induced lung cancer in animal models. In inflammation-related lung cancer, CHI3L1 is a downstream product of the inflammation-associated transcription gene *STAT3*, which is substantially expressed before tumor formation. STAT3 promotes the proliferation and growth of lung cancer cells and inhibits their apoptosis following tumor formation [138]. P53 can be directly targeted for degradation and ubiquitination by the E3 ubiquitin ligase. CHI3L1 inhibits the activity of p53 and its target proteins through its chitin-binding domain, induces p53 ubiquitination in lung cancer cells, and promotes the occurrence of lung cancer [139]. BRP-39 expression significantly increased in mice exposed to cigarette smoke after only two weeks, and increased CHI3L1 expression was also readily observed in smokers [140]. In mice exposed to cigarette smoke, BRP-39−/− animals exhibited more numerous apoptotic alveolar macrophages and neutrophils than their wild-type counterparts; this suggests that BRP-39 regulates disease progression by inhibiting inflammatory cell apoptosis during inflammation induced by cigarette smoke [140]. Lee et al. further investigated the mechanism by which BRP inhibits inflammatory cell apoptosis and found elevated expression levels of the apoptosis-inhibiting molecule Faim3, PKB/AKT activation, stimulated dendritic cell activation, and selective induction of macrophage activation and accumulation in mice under supraphysiological levels of BRP-39. Additionally, BRP-39 promotes the initiation and effector stages of Th2 inflammation and tissue fibrosis in the lung [92].

COPD is a greater risk factor for lung cancer than age or smoking level [141]. Although smokers experience inflammation in the lungs, it is more pronounced in patients with COPD, and chronic inflammation is critical to the development of COPD [142]. The infiltration of inflammatory cells, airway remodeling caused by persistent airway inflammation, and airway mucus hypersecretion are characteristic features of COPD [143]. Mucus is a moving barrier in the airways that can trap foreign particles, poisons, and pathogens. However, excessive mucus secretion can lead to airway obstruction [144]. The overproduction of mucin 5AC (MUC5AC) in a human bronchial epithelial cell line (16HBE) increased in a time- and dose-dependent manner with CHI3L1 stimulation. As a potent activator of protease-activating receptor 2 (PAR2), CHI3L1 appears to regulate MUC5AC secretion by epithelial cells through PAR2 activation, which in turn leads to ERK and NF-κB phosphorylation [145]. In addition, CHI3L1 induced the production of the inflammatory factors IL-8, RANTES, eotaxin, and IL-6 by activating the phosphorylation of transcription factors NF-κB and ERK/JNK. This process causes bronchial smooth muscle cells to lose polarity and gain migration ability, accelerates airway remodeling and fibrosis formation, and further promotes airway inflammation and remodeling in asthma [146].

The MAPK pathway, which mediates inflammation, is also involved in CHI3L1-mediated fibroblast proliferation, differentiation, and collagen synthesis [147]. Idiopathic pulmonary fibrosis (IPF) is a fatal chronic disease characterized by tissue fibrosis and diffuse interstitial inflammation. The incidence of lung cancer in patients with IPF is 2.7–48%, which is higher than that in patients with lung carcinoma without IPF [135]. The recruitment of circulating monocytes to the lungs increases the severity of fibrosis as they differentiate toward macrophage subtypes in response to microenvironmental changes caused by the gradual progression of lung fibrosis; M2 macrophages increase fibrosis severity [65]. In patients with IPF, the TGF-β/SMAD signaling pathway is one of the main mechanisms that induce fibroblast proliferation and differentiation, alveolar epithelial cell apoptosis, and imbalances in extracellular matrix synthesis [135]. The expression of BRP-39 increased in the lung tissue in an animal model of pulmonary fibrosis, accompanied by increased TGF-β1 expression and pronounced signs of pulmonary fibrosis [148]. In lung bronchial epithelial cells (BEAS-2B), CHI3L1 induced the TGF-β1/Smads pathway and increased cell proliferation and migration capacity, while inducing elevated concentrations of the proinflammatory factors IL-8, IL-4, and IL-6 [45]. IL-18, a product of inflammatory vesicle activation during lung fibrosis, is associated with poorer fibrosis progression [149, 150]. IL-18 inhibits cytotoxic cells to regulate alveolar septal destruction and airway fibrosis, and, notably, IL-18-induced progression of pulmonary fibrosis was alleviated in the absence of BRP-39, a downstream effector of IL-18 [146].

### CHI3L1 and hepatocellular carcinoma and gallbladder cancer

Sarcopenia is a prominent symptom in patients with advanced liver disease. CHI3L1 is overexpressed and secreted via TNF-α/TNF-R1 signaling to protect muscles from inflammatory factors such as lipopolysaccharide or TNF-α. It also regulates oxidative stress, the abnormal accumulation of lipid peroxide, as well as the expression of aggressive proteins, such as MMP2 and MMP7, ultimately playing a role in promoting carcinogenesis [151]. In addition, the increased risk of hepatocellular carcinoma is dependent on the progression of liver fibrosis [152]. TGF-β signaling is associated with tissue fibrosis, playing an oncogenic role before cancer formation, and its dysregulation can lead to tumorigenesis [153]. The correlation between *CHI3L1* and *TGF-β* mRNA in the liver and serum of patients with hepatitis C demonstrates that serum CHI3L1 and TGF-β originate from the liver and that serum CHI3L1 levels are positively correlated with TGF-β levels [154].

With the popularization of the hepatitis virus vaccine, the global hepatitis virus infection rate has decreased; however, the prevalence of metabolic-associated fatty liver disease (MAFLD) is increasing with the improvement in living standards [155]. In vitro, CHI3L1 leads to the activation of hepatic stellate cells and the expression of profibrotic mediators through IL-13αR2. Moreover, the secretion of inflammatory factors and chemokines increases in MAFLD mice in the presence of BRP-39 [156]. The recruitment of inflammatory cell aggregates is accompanied by CHI3L1-mediated inhibition of hepatic macrophage apoptosis, achieved through the suppression of Fas expression and autocrine activation of Akt signaling, leading to progressive liver fibrosis [157]. In advanced liver disease, the inflammatory factor TNF-α exerts a pro-carcinogenic effect through CHI3L1, leading to the accumulation of lipid peroxide [151], whereas in MAFLD, CHI3L1 induces an increase in lipid content in hepatocytes through the AKT, glycogen synthase kinase (GSK), and ERK pathways [158]. In the section on inflammatory bowel disease and colorectal cancer, we mentioned that CHI3L1 promotes the internalization of *S*. *typhimurium* in host cells, leading to inflammatory cell infiltration, IL-6 and IL-8 production, prolongation of inflammatory processes, and increased carcinogenicity in intestinal inflammation. In addition to increased susceptibility to colorectal cancer, the chronic presence of *S. typhimurium* in the gallbladder due to infection with the bacterium is also a risk factor for the development of gallbladder cancer [159]. Gallbladder cancer growth differentiation factor 15 (GDF15), a member of the TGF-β superfamily, is a multifunctional molecule that plays a role in inflammatory and apoptotic pathways in disease [160]. CHI3L1, derived from M2 macrophages, induces gallbladder cancer cells to secrete GDF15 and promotes programmed cell death protein 1 (PD-1) expression via P13K/AKT signaling to inhibit the cytotoxic effect of T cells, leading to the immune escape of tumor cells [161].

## Application of anti-inflammatory therapies targeting CHI3L1

Strategies that target CHI3L1 for the treatment of cancer recently have recently demonstrated progress in preclinical studies. Various CHI3L1 binding agents or inhibitors, including natural compounds such as chitin, ebractenoid F, and G721-0282, can inhibit block its mediated signaling pathway activity, thereby reducing or even inactivating it [48, 162–164]. The compound K284 with a CHI3L1 chitin-binding domain can effectively inhibit the binding of CHI3L1 to its receptor, IL-13Rα2, to suppress lung cancer metastasis [53]. Interfering RNA targeting CHI3L1 inhibits the drug resistance, proliferation, and invasion of cancer cells [165, 166]. Additionally, anti-CHI3L1 antibodies may enhance the association between syndecan-1 and N-cadherin by binding to the heparan sulfate chain on the syndecan-1 ectodomain. This results in the dissociation of N-cadherin from β-catenin, thereby resulting in the loss of the function of N-cadherin function in the membrane. Consequently, mural cell-mediated vascular stability is compromised, angiogenesis is reduced, and tumor development is inhibited owing to inadequate blood perfusion, nutrition, and oxygen delivery during tumor growth [167].

With the increasing application of immunotherapy in tumor treatment, the role of CHI3L1 in tumor immune escape has received considerable attention. CHI3L1 suppresses the antitumor response of NK cells and potentially other effector cells (e.g., CD8 + T cells), allowing tumor cells to escape recognition and attack by the immune system and maintaining inflammatory cell recruitment.

The role of PD-1 in cancer immune evasion has been well established. The interaction between PD-1 and programmed death-ligand 1 (PD-L1) initiates T cell programmed death, leading to immune evasion by tumor cells [168]. CHI3L1 can stimulate PD-L1 expression in lung melanoma cells, thereby inhibiting the antitumor activity of effector T cells. Bispecific antibodies that target CHI3L1 and PD-1 can effectively enhance the activity of CD8 cytotoxic T cells in the tumor microenvironment and induce the expression of the tumor suppressor gene PTEN in tumor cells, thereby playing a synergistic antitumor effect [169]. Yu et al. [170] found that anti-CHI3L1 antibodies decrease the phosphorylation and nuclear localization of STAT6 in macrophages. The reduction in STAT6 activation primarily affects M2 macrophage polarization but not that of M1-like macrophages. Consequently, anti-CHI3L1 antibodies can inhibit lung tumor migration by suppressing M2 polarization. *P53* is one of the most extensively studied tumor suppressor genes. CHI3L1 can promote the proliferation and metastasis of colon cancer cells by downregulating p53 and upregulating EGFR expression. Notably, it can also enhance sensitivity to cetuximab anticancer therapy, suggesting that CHI3L1 may play a pivotal role in guiding personalized cetuximab-based treatments [171].

Since CHI3L1 plays a key role in the development of chronic inflammatory diseases and subsequent inflammation-mediated cellular carcinogenesis, therapeutic strategies that target CHI3L1 are a hotspot of current research and are expected to provide new avenues and approaches for tumor therapy. Considering that CHI3L1 has numerous receptors and participates in complex signaling, increased research is required to identify effective inhibitory pathways and provide reliable data against CHI3L1-driven inflammation-associated cancers.

## Data Availability

No datasets were generated or analysed during the current study.
